# Melanoma Metastasis to a Pituitary Neuroendocrine Tumor: A Case Report and Literature Review

**DOI:** 10.7759/cureus.117965

**Published:** 2026-10-15

**Authors:** Alexey N Shkarubo, Ilia V Chernov, Liudmila V Shishkina, Daniil A Konyashin, Suzanne A Galstyan, Ivan V Chekhonin, Artem D Donskoy

**Affiliations:** 1 Neurooncology, Burdenko Neurosurgical Center, Moscow, RUS; 2 Neurosurgery, Sechenov University, Moscow, RUS; 3 Neuropathology, Burdenko Neurosurgical Center, Moscow, RUS; 4 X-ray and Radioisotope Diagnostic Methods, Burdenko Neurosurgical Center, Moscow, RUS

**Keywords:** malignant melanoma, pituitary metastasis, pituitary neuroendocrine tumor, transsphenoidal surgery, tumor-to-tumor metastasis

## Abstract

We report a 44-year-old man with a non-functioning pituitary macroadenoma diagnosed approximately 10 years before the current presentation. He subsequently developed metastatic amelanotic melanoma originating from a cutaneous lesion of the right auricle, with jejunal metastases. After disease progression despite immunotherapy and chemotherapy, he presented with visual disturbances and panhypopituitarism. Magnetic resonance imaging demonstrated a 28 × 22 × 30 mm endo-suprasellar mass compressing the optic chiasm. The patient underwent endoscopic transnasal transsphenoidal resection. Histopathological and immunohistochemical examination identified two tumors of distinct histogenesis within the same specimen: a pituitary adenoma and metastatic melanoma. The melanoma component was positive for Melan-A, HMB-45, SOX10, and S-100, confirming melanoma metastasis to the pituitary adenoma and establishing tumor-to-tumor metastasis. Postoperatively, the patient's visual function improved, and he was discharged on postoperative Day 5. This case demonstrates the diagnostic value of thorough histopathological and immunohistochemical evaluation when the clinical behavior of a previously diagnosed sellar lesion changes in a patient with systemic malignancy. Surgical decompression improved visual function and provided a definitive tissue diagnosis, supporting coordinated multidisciplinary management.

## Introduction

Secondary neoplastic involvement of the brain is one of the challenging areas of neurosurgery [1]. Metastasis of tumors to the brain reflects the terminal stage of oncological disease, which is why patients in severe condition often seek neurosurgical care [1]. The most common sources of CNS metastases are tumors of the breast, lung, and gastrointestinal tract, with their preferred sites being the cerebral hemispheres (80%), cerebellum (15%), and brainstem (5%) [1]. In no more than 5% of cases, the hypothalamic-pituitary system, and specifically the pituitary gland, is subject to secondary involvement [2]. In the available literature, we were able to find only 657 such cases [2,3]. A case-based situation is metastasis to a pre-existing pituitary macroadenoma [4], and such an event is extremely rare in melanomas [4]. Currently, only four such cases have been reported in the world literature, and we present the fifth [4-7].

## Case presentation

A 44-year-old man was admitted to a neurosurgical center with progressive visual impairment and headaches. The day of admission was designated as Day 1. His initial symptoms had developed approximately 10 years before Day 1. At that time, brain magnetic resonance imaging (MRI) revealed a small intrasellar lesion radiologically consistent with a pituitary adenoma. The original MRI images from that examination were not available for review. Hormonal evaluation indicated that the lesion was non-functioning. Given its small size, clinical and radiological surveillance without active treatment was recommended.

During follow-up for the pituitary lesion, one year after its initial detection, the patient underwent laser removal of a pigmented skin lesion on the right auricle. Initial histopathological examination classified the lesion as a nevus. The patient was subsequently lost to follow-up and remained without medical surveillance for six years.

Approximately two years before Day 1, an abdominal MRI performed during a routine medical examination revealed lesions suspicious for neoplastic involvement of the jejunal wall, jejunal mesenteric lymph nodes, and left intra-abdominal fascia. Shortly thereafter, the patient underwent laparoscopic segmental resection of the jejunum with a side-to-side anastomosis at his local hospital. Histopathological examination revealed metastatic amelanotic epithelioid melanoma. Immunohistochemistry demonstrated tumor-cell positivity for Melan-A, HMB-45, SOX10, S100, and tyrosinase and negativity for pancytokeratin (PanCK), CK7, CK20, synaptophysin, and chromogranin A. Subsequent review of the histological slides from the previously removed right auricular lesion resulted in revision of the original diagnosis to epithelioid melanoma.

Pembrolizumab was initiated approximately two months after the intestinal surgery. Treatment was discontinued three months later because of disease progression. Positron emission tomography/computed tomography (PET/CT), performed approximately two weeks after treatment discontinuation, demonstrated an increased number of metabolically active metastatic lesions involving the jejunum, left iliac intra-abdominal fascia, and jejunal mesenteric lymph nodes. The patient subsequently received four cycles of Nurdanti over approximately two months, resulting in decreased size and metabolic activity of the metastatic lesions. Prolgolimab was initiated approximately three weeks after the final cycle and continued for approximately 13 months. During treatment, follow-up PET/CT demonstrated further disease progression, with new metastases in the supradiaphragmatic adipose tissue and soft tissues.

During Month 11 of prolgolimab therapy, the patient noticed decreased visual acuity and loss of the temporal visual fields. Brain MRI demonstrated progressive enlargement of the previously identified sellar lesion. During hospitalization, ophthalmological examination confirmed temporal visual-field constriction, which was more pronounced on the left. With +1.5-diopter spherical correction, decimal visual acuity was 1.0 in the right eye and 0.8 in the left eye. Laboratory findings were consistent with panhypopituitarism, including secondary hypogonadism, secondary adrenal insufficiency, and hypothyroidism. The testosterone concentration was 2.08 nmol/L (reference range: 8.33-30.19 nmol/L), cortisol was 58.2 nmol/L (reference range: 101-536 nmol/L), and free thyroxine was 6.54 pmol/L (reference range: 9-19 pmol/L). Apart from the visual impairment, neurological examination revealed no abnormalities.

Contrast-enhanced brain MRI revealed a well-circumscribed, ovoid endo-suprasellar mass measuring approximately 28 × 22 × 30 mm, with laterosellar extension and compression of the optic chiasm (Figure 1). The lesion had a heterogeneous internal architecture. Its peripheral portions were hyperintense on T2-weighted images and isointense to cerebral gray matter on T1-weighted images, with avid contrast enhancement. A deeper, eccentrically located left-sided component was iso- to hypointense on both T1- and T2-weighted images and showed less intense enhancement. An additional small focus with similar signal characteristics was present along the superior margin, and a minute cystic component was observed centrally. The sella turcica was expanded. The anterior cerebral artery complex was closely apposed to the superior surface of the lesion, and the cavernous segments of the internal carotid arteries were located lateral to the mass. The pituitary stalk could not be reliably identified because of compression.

**Figure 1:**
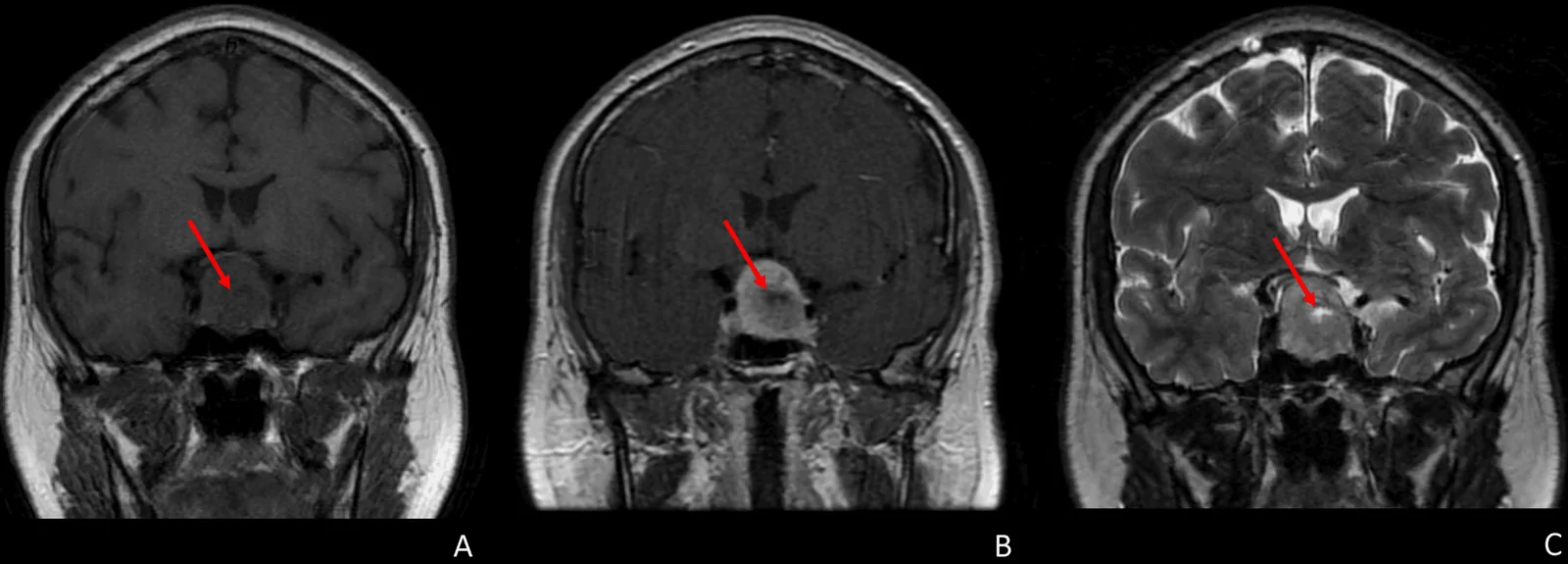
Preoperative MRI. Coronal non-enhanced T1-weighted image (A), enhanced T1-weighted image (B), and T2-weighted image (C) demonstrate a heterogeneous endo-suprasellar mass compressing the optic chiasm. The arrows indicate the distinct intratumoral component that raised suspicion for metastatic involvement.

During the current admission, the patient underwent endoscopic endonasal transsphenoidal resection of the endo-suprasellar tumor, followed by reconstruction of the skull base defect using autologous tissue. The initially encountered tumor component was grayish, moderately vascular, and moderately firm. After removal of tissue grossly resembling a pituitary adenoma, a visually distinct component with a different color and consistency was identified. This component contained minute calcifications and intraoperatively resembled a craniopharyngioma. Resection was performed using suction and curettes under visualization with 0°, 30°, and 45° endoscopes. Macroscopically complete resection was achieved within the visible surgical margins.

Immediate postoperative computed tomography demonstrated minimal pneumocephalus and fragments of hemostatic material in the operative bed, without evidence of surgical complications. The postoperative course was uneventful. Within one week, visual function improved, and the left temporal visual-field constriction resolved. The patient was discharged for outpatient follow-up with local specialists on postoperative Day 5.

At postoperative Month 3, contrast-enhanced MRI demonstrated no residual sellar tumor (Figure 2).

**Figure 2:**
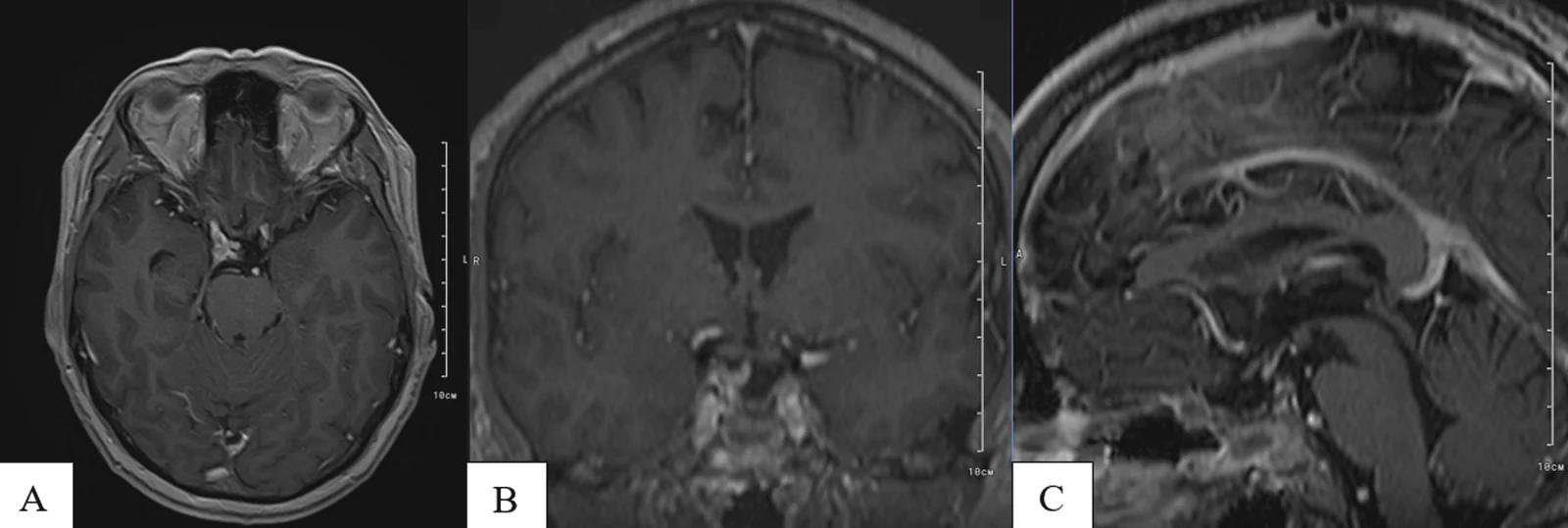
Contrast-enhanced T1-weighted magnetic resonance images obtained at postoperative Month 3. (A) Axial, (B) coronal, and (C) sagittal views demonstrate no residual sellar tumor.

Whole-body PET/CT performed at postoperative Month 3 demonstrated progression of systemic metastatic disease in the subcutaneous and soft tissues, with no persistent metabolically active lesion in the sellar region (Figure 3).

**Figure 3:**
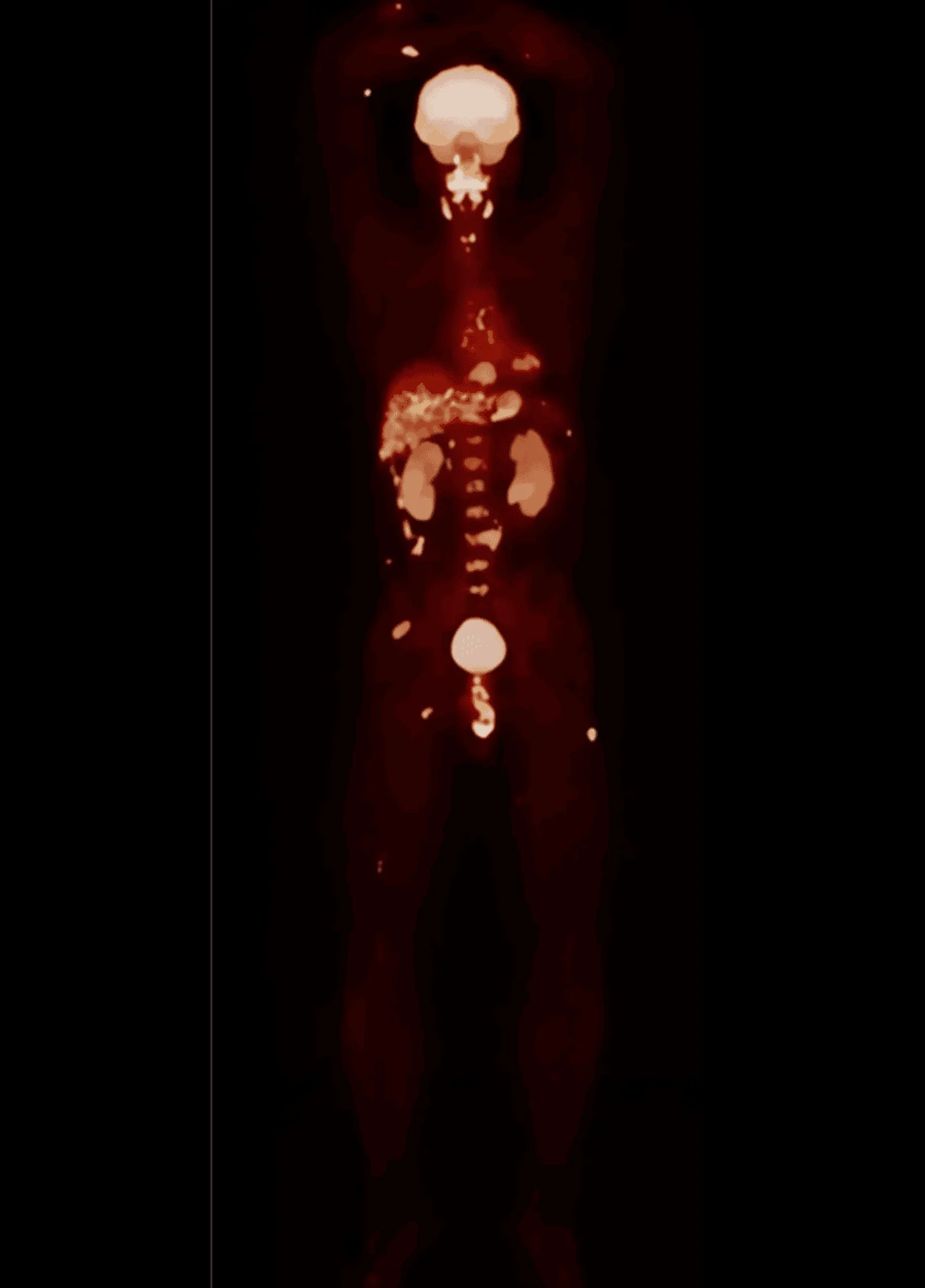
Whole-body positron emission tomography/computed tomography obtained at postoperative Month 3. The examination demonstrates progression of systemic metastatic disease in the subcutaneous and soft tissues, with no persistent metabolically active lesion in the sellar region.

Histopathological examination

Histopathological examination revealed two morphologically distinct tumor components. The first consisted of relatively monomorphic small-to-medium-sized cells with faintly eosinophilic cytoplasm, ovoid nuclei, low mitotic activity with occasional mitoses, and a predominantly perivascular growth pattern. The second component consisted of large pleomorphic epithelioid cells with marked nuclear atypia, brisk mitotic activity, and foci of necrosis. The two components were directly juxtaposed (Figure 4).

**Figure 4:**
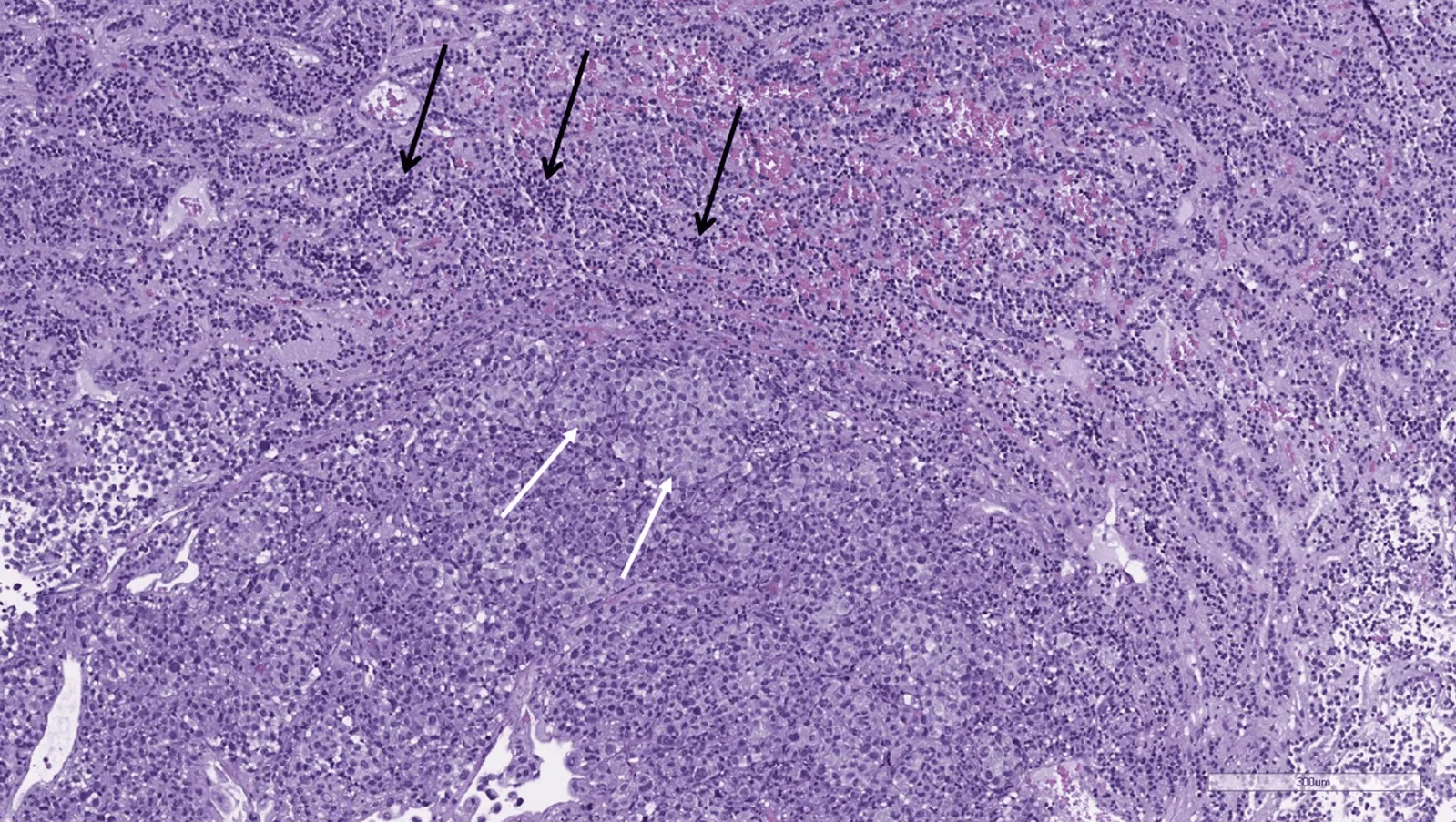
Hematoxylin and eosin-stained section demonstrating juxtaposed components of a pituitary neuroendocrine tumor and metastatic melanoma (original magnification ×100). Black arrows indicate the monomorphic small-cell pituitary component, and white arrows indicate the pleomorphic epithelioid melanoma component.

Immunohistochemical examination demonstrated synaptophysin expression in the small-cell pituitary component, with complete absence of staining in the large epithelioid cells. PanCK (AE1/AE3) was focally expressed in the small-cell component and absent from the epithelioid component. Conversely, the large epithelioid cells demonstrated nuclear and cytoplasmic S100 expression and cytoplasmic Melan-A expression, whereas the small-cell component was negative for both markers. The Ki-67 proliferation index reached approximately 30% in the large-cell component and did not exceed 5% in the small-cell component (Figure 5).

**Figure 5:**
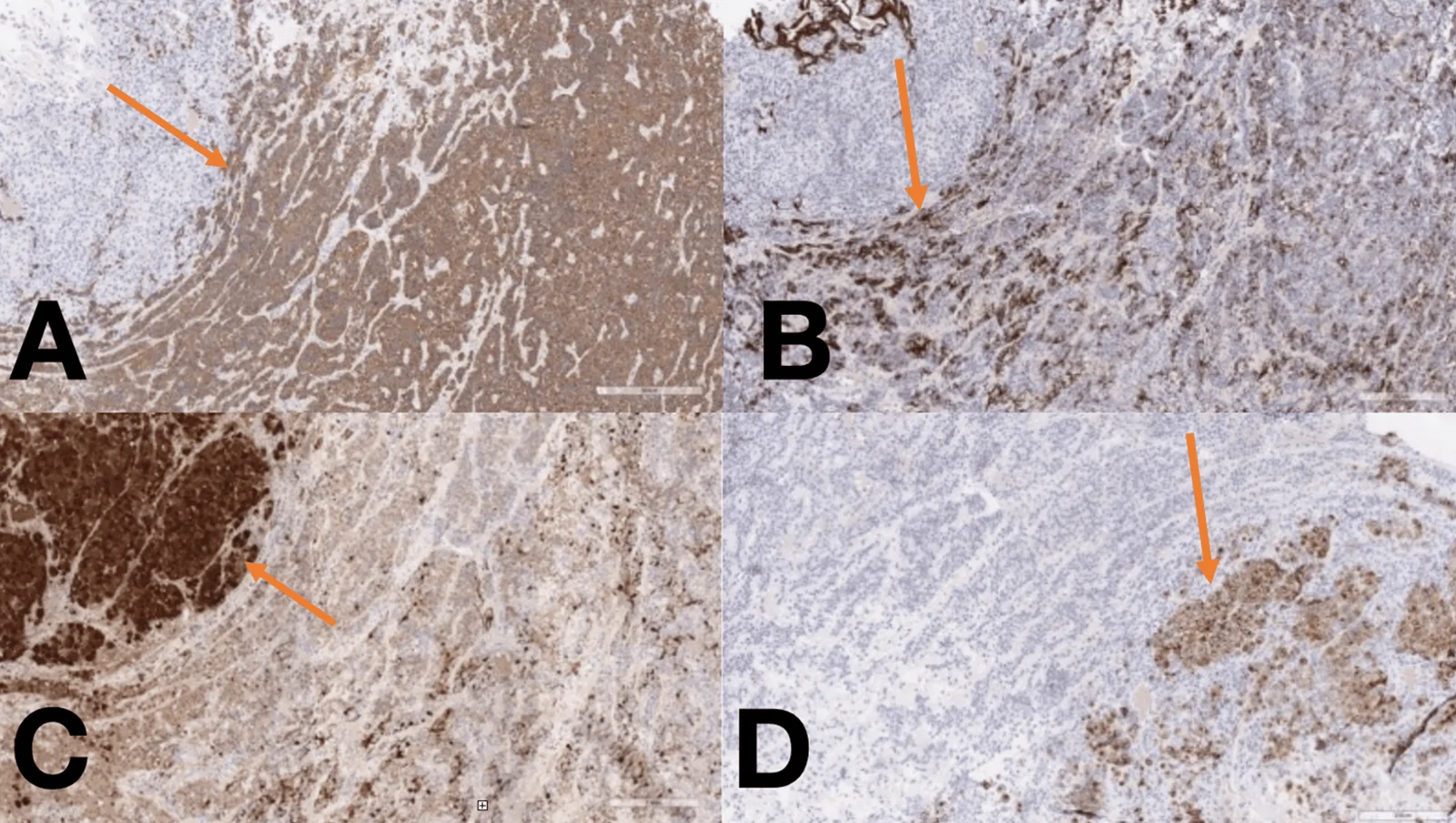
Immunohistochemical differentiation of the pituitary tumor and metastatic melanoma components (original magnification ×100). (A) Synaptophysin is expressed in the small pituitary tumor cells and absent from the large epithelioid melanoma cells; the orange arrow marks the interface between the two components. (B) PanCK (AE1/AE3) is focally expressed in the small pituitary tumor cells and absent from the epithelioid melanoma cells; the orange arrow indicates focally stained pituitary tumor cells adjacent to the unstained melanoma component. (C) S100 demonstrates diffuse nuclear and cytoplasmic expression in the epithelioid melanoma cells and is absent from the small pituitary tumor cells; the orange arrow marks the interface between the S100-positive and S100-negative components. (D) Melan-A demonstrates cytoplasmic expression in the large epithelioid melanoma cells and is absent from the small pituitary tumor cells; the orange arrow indicates a cluster of Melan-A-positive melanoma cells adjacent to the unstained pituitary tumor component.

The combined morphological and immunohistochemical findings confirmed the presence of two neoplasms of distinct histogenesis within the same surgical specimen: a pituitary adenoma and metastatic melanoma. These findings established tumor-to-tumor metastasis of melanoma to a pre-existing pituitary adenoma.

## Discussion

In neurosurgical practice, a patient with melanoma metastasis to a pituitary macroadenoma is a rare occurrence, and therefore a high index of suspicion for such a combination is not always present. A thorough analysis of the patient's medical history and imaging findings may help to suspect secondary pituitary involvement [3,8,9].

It is known that cutaneous melanoma ranks 17th in incidence among malignant neoplasms [8], most commonly metastasizing to the lungs, liver, and brain, where it can implant in virtually any region [8,10,11]. Unfortunately, such metastatic spread currently carries a very poor prognosis, as despite all modern treatment modalities, median survival is four to six months [10,11].

The development of a melanoma metastasis within the pituitary gland represents an exceedingly rare manifestation of the disease, with secondary lesions predominantly located in the neurohypophysis [3,8,9]. This tropism has been attributed in the literature to the richer blood supply of the posterior pituitary lobe relative to the anterior lobe. Furthermore, implantation in the adenohypophysis requires tumor cells to traverse the microvascular network connecting the two lobes, thereby posing a more formidable natural barrier [3,8].

A distinct clinical scenario is represented by metastasis into a pre-existing pituitary tumor [4-6]. Given that pituitary adenomas are relatively common (approximately 25-89 cases per 100,000 population), melanoma metastasis to such an adenoma does not appear unfeasible but occurs only as a casuistic rarity [12]. Several theories have been proposed in the literature to explain these events. One postulates elevated levels of alpha-melanocyte-stimulating hormone (α-MSH) and its receptor, which stimulate melanin production and, consequently, facilitate the implantation of melanoma cells into a pituitary adenoma [5,6].

To date, only four cases of melanoma metastasis to a pre-existing pituitary tumor have been reported in the world literature (Table 1) (three males, one female; mean age: 67.3 years) [4-7]. The cases reported in the literature involved large sellar masses, with visual deterioration and suspected pituitary apoplexy reported as the main indications for surgery [4-6]. In only one of these cases was the presence of two distinct neoplasms suspected preoperatively [4-6]. In our case, the MRI findings and medical history also raised suspicion of a metastasis within the pituitary adenoma. Nevertheless, the surgical strategy remained unchanged and consisted of radical resection of the pituitary adenoma along with the embedded metastasis, which was noted to be of firmer consistency - a finding likewise reported by colleagues describing similar cases [4-6]. The endocrine profile did not differ from that observed in patients with pituitary adenomas and required identical hormone replacement therapy in the postoperative period [3-6].

**Table 1:** Melanoma metastasis to a pre-existing pituitary tumor. BRAF - B-Raf proto-oncogene, serine/threonine kinase; F - female; M - male; ACTH - adrenocorticotropic hormone; FT3/FT4 - free triiodothyronine/thyroxine; PitNET - pituitary neuroendocrine tumor

Case	Age/sex	Host pituitary tumor	Primary melanoma site and histological subtype	Distribution of metastatic disease	Clinical presentation prompting sellar surgery	BRAF mutation status	Systemic antitumor therapy received	Outcome/follow-up
Yang et al., 2017 [5]	62/F	Prolactinoma	Left heel cutaneous melanoma; specific histological subtype not reported.	Suspected liver metastases and hepatic hilar, retroperitoneal and inguinal nodal disease; subsequent cutaneous metastases in the lower limbs and groins.	Progressive visual loss with bitemporal hemianopia and headache; hyperprolactinemia.	Not reported	Not reported; further antitumor treatment was declined after sellar surgery.	Visual fields and headache improved; endocrine indices normalized with replacement. Alive at 22 months with progressive disease and severe neurological/functional decline.
Lamorie-Foote et al., 2021 [4]	64/M	Nonfunctioning gonadotroph macroadenoma	Primary anatomical site not specified; acral lentiginous melanoma.	Abdominal and pelvic lymph nodes, lungs and liver; new bone metastases at 3 months postoperatively.	Hypopituitarism with mild hyperprolactinemia; no diabetes insipidus, headache or visual deficit. Tumor enlargement and new intratumoral hemorrhage prompted surgery, without clinical apoplexy.	Not reported	Ipilimumab, pembrolizumab and ibrutinib during the treatment course.	Neurologically intact after surgery; hypopituitarism persisted. Disease progression at 3 months; died soon afterward from an unrelated accident.
Jung et al., 2007 [7]	Mid-70s/M	Pituitary oncocytoma (hormone negative)	Left great toe, subungual; acral lentiginous melanoma.	Biopsy-proven left inguinal nodal metastasis; suspected mediastinal, porta-hepatis, para-aortic and bilateral inguinal nodal disease and a left renal-hilar lesion.	Progressive visual loss; hypoglycemia and a fall with loss of consciousness; suspected pituitary apoplexy. Mild hyperprolactinemia, hypothyroidism and partial ACTH deficiency.	Not reported	Not reported	Died 1 month after surgery; postoperative visual outcome not reported.
Ramos et al., 2017 [6]	67/M	Nonfunctioning pituitary adenoma	Skin of the back; nodular melanoma, Clark level IV.	New left frontal opercular lesion at 2 months after sellar surgery; extracranial metastatic sites not reported.	Sudden headache, vomiting and reduced left-eye acuity; presumed pituitary apoplexy. Pre-existing hypopituitarism treated with levothyroxine, hydrocortisone and testosterone.	Not reported	Not reported	Initial clinical improvement; sellar regrowth and a new frontal lesion at 2 months. Subsequently died;
Current case	44/M	Nonfunctioning PitNET	Right auricle skin; epithelioid morphology, with amelanotic epithelioid metastases.	Jejunum, mesenteric lymph nodes, intra-abdominal fascia, supradiaphragmatic fat, subcutaneous and other soft tissues.	Progressive visual impairment with temporal field loss (left predominant) and headache; panhypopituitarism, including secondary adrenal insufficiency, hypogonadism and hypothyroidism.	Reported negative*	Pembrolizumab (3 cycles), followed by nurulimab + prolgolimab (Nurdati; 4 cycles), then prolgolimab monotherapy. Subsequent chemotherapy; regimen not specified.	Visual function improved; no residual sellar tumor at 3 months. Systemic disease progressed; alive at 4 months.

Given the presence of stage IV oncological disease, these patients required oncological follow-up and, understandably, had predictably poorer outcomes. In all reported cases, the tumor-to-tumor phenomenon was identified against a background of multiple melanoma metastases. Moreover, secondary lesions in other organs led to deterioration of the patients' somatic status, resulting in a median postoperative survival of only three months. Of the five cases (four previously published and one described in the present report), two patients remain alive to date [4-7].

According to a historical randomized study, chemotherapy that included temozolomide yielded a median survival of approximately 7.7 months [13]. At that time, immunotherapy and molecularly targeted therapy were rarely used. Modern systemic therapy includes immunotherapy with immune checkpoint inhibitors and, in patients with a confirmed B-Raf proto-oncogene, serine/threonine kinase (BRAF) V600 mutation, combined molecularly targeted therapy with BRAF and mitogen-activated protein kinase kinase (MEK) inhibitors. For example, the combination of nivolumab and ipilimumab increased three-year survival to 71.9%; however, in patients with neurologically significant metastases, the prognosis remains poor: three-year survival with the same therapy is almost halved (36.6%) [9,10,13-19,20]. Combining systemic therapy with radiotherapy may also improve overall and progression-free survival, although this is associated with a risk of radiation necrosis and high toxicity [14-17]. The preferred option is high-precision radiotherapy, which minimizes complications and adverse effects on healthy tissues, which is particularly important when the pituitary gland is involved [15-19].

## Conclusions

Melanoma metastasis to a pre-existing pituitary adenoma is an exceptionally rare tumor-to-tumor phenomenon that may not be readily suspected in clinical practice. Unexpected progression of a previously stable sellar lesion and changes in its MRI signal characteristics may raise suspicion of metastatic involvement. In the present case, despite advanced systemic disease, surgical treatment resulted in visual recovery and local tumor control without perioperative complications. The role and outcomes of systemic therapy in this setting require further investigation given the limited number of reported cases. Future reports should document disease stage and BRAF mutation status to better inform systemic treatment selection and prognostic assessment. A multidisciplinary approach involving neurosurgeons, radiologists, and oncologists, including specialists in systemic anticancer therapy, remains central to patient management.
